# Establishment and validation of a nomogram model for riskprediction of hepatic encephalopathy: a retrospective analysis

**DOI:** 10.1038/s41598-023-47012-z

**Published:** 2023-11-09

**Authors:** Chun Yao, Liangjiang Huang, Meng Wang, Dewen Mao, Minggang Wang, Jinghui Zheng, Fuli Long, Jingjing Huang, Xirong Liu, Rongzhen Zhang, Jiacheng Xie, Chen Cheng, Fan Yao, Guochu Huang

**Affiliations:** 1 First Affiliated Hospital of Guangxi University of Chinese Medicine, 89-9 Dongge Road, Nanning, 530001 Guangxi People’s Republic of China; 2 Guangxi University of Chinese Medicine, Nanning, 530001 Guangxi People’s Republic of China

**Keywords:** Risk factors, Gastrointestinal diseases

## Abstract

To establish a high-quality, easy-to-use, and effective risk prediction model for hepatic encephalopathy, to help healthcare professionals with identifying people who are at high risk of getting hepatic encephalopathy, and to guide them to take early interventions to reduce the occurrence of hepatic encephalopathy. Patients (n = 1178) with decompensated cirrhosis who attended the First Affiliated Hospital of Guangxi University of Chinese Medicine between January 2016 and June 2022 were selected for the establishment and validation of a nomogram model for risk prediction of hepatic encephalopathy. In this study, we screened the risk factors for the development of hepatic encephalopathy in patients with decompensated cirrhosis by univariate analysis, LASSO regression and multifactor analysis, then established a nomogram model for predicting the risk of getting hepatic encephalopathy for patients with decompensated cirrhosis, and finally performed differentiation analysis, calibration analysis, clinical decision curve analysis and validation of the established model. A total of 1178 patients with decompensated cirrhosis who were hospitalized and treated at the First Affiliated Hospital of Guangxi University of Chinese Medicine between January 2016 and June 2022 were included for modeling and validation. Based on the results of univariate analysis, LASSO regression analysis and multifactor analysis, a final nomogram model with age, diabetes, ascites, spontaneous peritonitis, alanine transaminase, and blood potassium as predictors of hepatic encephalopathy risk prediction was created. The results of model differentiation analysis showed that the AUC of the model of the training set was 0.738 (95% CI 0.63–0.746), while the AUC of the model of the validation set was 0.667 (95% CI 0.541–0.706), and the two AUCs indicated a good discrimination of this nomogram model. According to the Cut-Off value determined by the Jorden index, when the Cut-Off value of the training set was set at 0.150, the sensitivity of the model was 72.8%, the specificity was 64.8%, the positive predictive value was 30.4%, and the negative predictive value was 91.9%; when the Cut-Off value of the validation set was set at 0.141, the sensitivity of the model was 69.7%, the specificity was 57.3%, the positive predictive value was 34.5%, and the negative predictive value was 84.7%. The calibration curve and the actual events curve largely overlap at the diagonal, indicating that the prediction with this model has less error. The Hosmer–Lemeshow test for goodness of fit was also applied, and the results showed that for the training set, χ^2^ = 1.237587, *P* = 0.998, and for the validation set, χ^2^ = 31.90904, *P* = 0.0202, indicating that there was no significant difference between the predicted and actual observed values. The results of the clinical decision curve analysis showed that the model had a good clinical benefit, compared with the two extreme clinical scenarios (all patients treated or none treated), and the model also had a good clinical benefit in the validation set. This study showed that aged over 55 years, complications of diabetes, ascites, and spontaneous bacterial peritonitis, abnormal glutamate aminotransferase and abnormal blood potassium are independent risks indicators for the development of hepatic encephalopathy in patients with decompensated cirrhosis. The nomogram model based on the indicators mentioned above can effectively and conveniently predict the risk of developing hepatic encephalopathy in patients with decompensated cirrhosis. The nomogram model established on this study can help clinical healthcare professionals to timely and early identify patients with high risk of developing hepatic encephalopathy.

## Introduction

Hepatic encephalopathy (HE) is an extremely serious complication of cirrhosis, and is the most common cause of death among various liver diseases. Hepatic encephalopathy has a great impact on patients' quality of life. Therefore, anticipatory interventions for those at risk for hepatic encephalopathy are particularly important. The personal experience of healthcare professionals is still the main basis for the assessment and identification of hepatic encephalopathy in clinical practice, making the diagnosis and treatment of hepatic encephalopathy significantly limited. If the risk of hepatic encephalopathy can be accurately predicted, if patients gets timely and early intervention, the progression of them getting hepatic encephalopathy can be stopped. However, the risk factors for the development of hepatic encephalopathy are not uniformly reported worldwide, the predictive ability of existed risk prediction models for hepatic encephalopathy is still unknown and failed to meet clinical needs. Therefore, this study screened the risk factors for the development of hepatic encephalopathy in patients with decompensated cirrhosis by univariate analysis, LASSO regression and multifactor analysis, and established a nomogram model for the prediction of risk of getting hepatic encephalopathy. The differentiation analysis, calibration analysis, clinical decision curve analysis and validation of the established model were also performed.

## Methods

### General information

**Figure 1:**
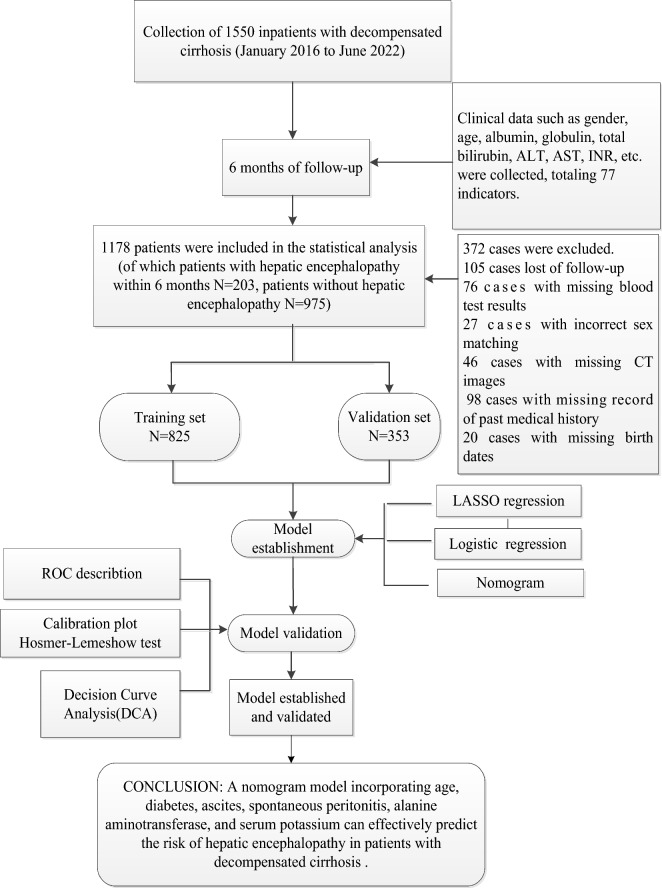
Flow diagram of the study.

This study is a retrospective cohort study. We identified 77 relevant indicators of decompensated cirrhosis. We collected data from a total of 1550 inpatients with decompensated cirrhosis who attended the First Affiliated Hospital of Guangxi University of Chinese Medicine from January 2016 to June 2022, and these patients were followed up and screened for six months. Of these 1550 patients, 372 were not included in the final analysis due to missing follow-up, missing or incorrect data, and/or missing indicators, the total missing rate was 24%. The final sample size evaluated for the modeling analysis was 1178. All patients received routine treatment, including treatment of the cause (antiviral, alcohol cessation, etc.); use of lactulose, probiotics, etc. to keep the patient's bowel movements soft with a frequency of 1–2 times per day during the follow-up period; and treatment of their complications, respectively. Among these 1178 patients, 203 patients developed hepatic encephalopathy within six months of follow-up and 975 patients did not develop hepatic encephalopathy. A flow diagram of the study design is shown in Fig. 1.

### Ethics statement

This study was reviewed and approved by The Ethics Committee of the First Affiliated Hospital of Guangxi University of Chinese Medicine (approval No.: 2022-080-02). All methods of this study were performed in accordance with the relevant guidelines and regulations. All subject researchers have pledged to follow the principle of confidentiality. All data and information collected were used for this study only. Informed consents were obtained from all subjects.

### Inclusion criteria

1. age ≥ 18 years.
2. Meet the diagnostic criteria of decompensated cirrhosis^1^.
3. No current overt hepatic encephalopathy.
4. No previous history of hepatic encephalopathy.

### Exclusion criteria

1. Missing more than 10% of data.
2. Imaging or pathological biopsy findings of liver cancer.
3. Combined severe systemic diseases or drug addicts who have difficulty quitting.
4. Pregnant or lactating women.
5. Combined pulmonary or other organ infections or gastrointestinal bleeding at admission.
6. Combined sepsis, shock from various causes, etc. at admission.
7. Combined acquired immunodeficiency virus (HIV) infection, syphilis spirochete infection.
8. Altered consciousness caused by concomitant psychiatric disease, metabolic encephalopathy, toxic encephalopathy and craniosynostosis.
9. History of laparotomy within 4 weeks prior to admission.
10. Record of alcohol consumption throughout the follow-up period.

### Statistical analysis

#### Data grouping

Random numbers were generated using R software, and patients with decompensated cirrhosis included in the study were randomly divided into a training set(70%) and a validation set(30%). The training set was set to construct a risk prediction model, and the validation set was set to verify the accuracy of the prediction model.

#### Statistical descriptions

Analysis of variance was performed for the 77 selected relevant indicators in the training and validation sets. Between-group comparisons were made between patients’ data in the hepatic encephalopathy and non-hepatic encephalopathy groups. Among these indicators, continuous variables such as white blood cell count (WBC), were compared between groups using one-sample independent *t* tests or Wilcoxon rank sum tests, expressed as Mean ± SD or Median (P25, P75). Categorical variables, such as smoking history, hypertension, diabetes mellitus, presence of gastrointestinal bleeding, presence of ascites, presence of cardiovascular disease, were compared between groups by Pearson's chi-square test or Fisher test for rates and expressed as frequencies (percentages). Statistically significant difference was set at *P* < 0.05.

#### Handling of missing values

Multiple interpolation of missing data was performed using SPSS software. Most of the traditional methods for handling missing values use median or mean for interpolation. Multiple interpolation deals with missing values by using other variables given in the dataset, fitting the missing values by iteration and pre-defined matrix construction models, and then using the fitted predicted values to multiply fill the missing values of this variable. This method gives a higher accuracy of the missing value alternatives.

#### Model establishment and demonstration

*(1) Determination of independent risk factors* Least absolute shrinkage and selection operator (LASSO) regression was performed using the "glmnet" package of R software. LASSO regression is a linear regression that avoids overfitting by imposing a penalty on the magnitude of the model coefficients. Some of the variables derived from the LASSO regression mightnot be significantly correlated with the results in the multi-factor logistic regression analysis.

*(2) Establishment of the model* After screening the predictor variables by LASSO regression, the variables with *P* < 0.1 were used as predictors, and the risk prediction model was constructed by binary logistic regression using the glm function in R software.

*(3) Presentation of the model* In order to visualize the weights of each predictor and to make the established risk prediction model more convenient and concise for clinical application, the "rms" package of R software was used to build a nomographic plot based on the results of the multi-factor logistic model by using the lrm function and nomogram function.

#### Evaluation and validation of the model

The risk prediction model for patients with decompensated cirrhosis getting hepatic encephalopathy built based on the training set was evaluated in terms of its discriminative efficacy, consistency test and clinical benefit. The model was validated in the validation set.

*(1) Evaluation of the model* ROC (receiver operator charteristics), area under curve (AUC), concordance index (C-index), sensitivity, specificity, positive predictive value (PPR), and risk prediction model were used to evaluate the model. The area under the ROC curve reflects the discriminative power of the model. The risk prediction model is considered to be having good discriminatory ability if the area under the ROC curve was greater than 0.7. On the contrary, when the area under the ROC curve was close or even equals to 0.5, the risk prediction model would be considered to be having low diagnostic value.

*(2) Validation of the model* In this study, we used the bootstrap resampling method, the Hosmer–Lemeshow test, the ROC curve, the area under the ROC curve, and the calibration curve to measure and validate the model. The clinical benefit of the model was evaluated using decision curve analysis.

This study used Excel software for data entry and SPSS 26.0 and R 3.6.3 software for statistical analysis of the data. All *P* values were two-sided tests, *P* < 0.05 indicates that the differences are statistically significant unless otherwise stated.

## Results

### Sample size estimation and general information of the included patients

We calculated the minimum sample size required for modeling in this study as 323 cases based on R^2^ and 344 cases based on C-index. According to the sample size estimation, we included 1178 patients with decompensated cirrhosis were finally included in the analysis, including 203 patients who developed hepatic encephalopathy within six months and 975 patients who did not. 849(72.1%) of the 1178 patients in the sample were male, 329(27.9%) were female. 585 patients(49.7%) were < 55 years of age, 593 patients(50.3%) were over 55 years of age. 195 patients(16.6%) had ascites; 410 patients(34.8%) had infection; 193 patients(16.4%) had diabetes mellitus; as shown on Table 1.

**Table 1:** General information of the included patients.

Variables (categorical variables)	N (%)/Mean ± SD/Median (P_25_, P_75_)	Variables (continuous variables)	N (%)/Mean ± SD/Median (P_25_, P_75_)
Occurrence of hepatic encephalopathy		HGB	126 [101;145]
No	975 (82.8%)	MCV	92.1 [85.3;97.0]
Yes	203 (17.2%)	MCH	30.7 [27.8;32.4]
Sex		PLT	126 [72.0;186]
Male	849 (72.1%)	MPV	9.90 [9.10;10.6]
Female	329 (27.9%)	PCT	0.12 [0.07;0.18]
Age (years)		K	3.80 [3.54;4.10]
< 55 years old	585 (49.7%)	Na	140 [138;142]
≥ 55 years old	593 (50.3%)	Cl	104 [102;106]
History of diabetes		P	1.01 [0.89;1.13]
No	985 (83.6%)	Mg	0.83 [0.76;0.90]
Yes	193 (16.4%)	Ca	2.18 [2.06;2.29]
History of smoking		TG	0.94 [0.70;1.36]
No	939 (79.7%)	CHOL	4.09 [3.30;4.98]
Yes	239 (20.3%)	HDL	1.17 [0.92;1.43]
History of alcohol consumption		APOA1	1.24 [0.98;1.49]
No	847 (71.9%)	APOB	0.78 [0.58;0.97]
Yes	331 (28.1%)	VLDL	0.43 [0.32;0.62]
History of cerebrovascular disease		LDL	2.33 [1.72;3.09]
No	1142 (96.9%)	LDH	188 [158;235]
Yes	36 (3.06%)	CK	107 [70.0;165]
History of cardiovascular disease		AHBDH	150 [125;188]
No	1115 (94.7%)	IgA	2.67 [1.91;3.36]
Yes	63 (5.35%)	IgG	15.6 [12.8;19.5]
Hypertension		IgM	1.22 [0.81;1.78]
No	980 (83.2%)	PT	14.4 [13.2;16.6]
Yes	198 (16.8%)	PTA	83.0 [64.0;99.0]
Ascites		INR	1.12 [1.01;1.35]
No	983 (83.4%)	APTT	41.0 [37.4;45.7]
Yes	195 (16.6%)	TT	17.5 [16.6;18.7]
Gastrointestinal bleeding		FIB	2.55 [1.96;3.14]
No	1154 (98.0%)	D-Dimer	0.55 [0.25;2.14]
Yes	24 (2.04%)	TP	66.7 [61.5;71.8]
Spontaneous bacterial peritonitis		ALB	37.9 [31.8;43.0]
No	1092 (92.7%)	GLO	28.2 [24.6;32.9]
Yes	86 (7.30%)	AVSG	1.38 [1.02;1.69]
infection		PA	156 [79.0;223]
No	768 (65.2%)	CHE	5768 [3425;8271]
Yes	410 (34.8%)	TBIL	20.3 [13.5;35.1]
Biliary tract disorders		DBIL	6.60 [4.10;14.5]
No	964 (81.8%)	IBIL	13.4 [9.10;20.3]
Yes	214 (18.2%)	TBA	18.4 [5.70;55.6]
Hepatorenal syndrome (HRS)		AKP	83.0 [65.0;117]
No	1166 (99.0%)	GGT	37.0 [23.0;75.8]
Yes	12 (1.02%)	ALT	29.0 [19.0;44.0]
Hepatitis B		AST	35.0 [27.0;52.8]
No	193 (16.4%)	ADA	19.0 [14.0;27.2]
Yes	985 (83.6%)	AFU	28.0 [23.0;36.0]
Hepatitis C		BUN	4.61 [3.70;5.78]
No	1127 (95.7%)	CREA	72.0 [61.0;84.0]
Yes	51 (4.33%)	UA	309 [245;380]
Variables (continuous variables)		CysC	0.88 [0.72;1.12]
WBC (× 10^9^/L)	5.10 [3.80;6.50]	CO2	24.2 [22.3;26.1]
NEUT	2.81 [2.10;3.80]	SOD	151 [126;174]
NEUP	58.5 (11.9)	CRP	10.0 [4.50;22.5]
LYT	0.14 [0.08;0.25]	Ammo	40.1 [23.4;57.0]
LYP	2.90 [1.60;4.90]	GLU	5.03 [4.50;5.97]
RBC(× 109/L)	4.26 [3.45;4.88]		

N (%): number of cases (percentage); Mean ± SD: mean ± standard deviation; Median: median; P25: 25th percentile; P75: 75th percentile.

### Comparsion of the training and validation sets

1178 patients were randomly divided into a training set and a validation set in the ratio of 70% and 30%, where the training set n = 826 and the validation set n = 352 (Table 2). The training set included 128 patients who heve hepatic encephalopathy, accounting for 15.5% of the group; the validation set included 75 patients who have hepatic encephalopathy, accounting for 21.3% of the group. Statistical analysis was performed on the general data, and the results shown that except for the neutrophil ratio (NEUT), absolute neutrophil value (NEUP), immunoglobulin M (IgM), and a-L-amyloidase (AFU), the differences between the two groups were not statistically significant (*P* > 0.05) for all variables. This indicates that the indicators in the training and validation sets are evenly distributed, which can effectively avoid the conclusion bias.

**Table 2:** Baseline table for training set-validation set grouping.

Variables	Total N = 1178	Validation set N = 352	Training set N = 826	*P* value
History of diabetes				0.406
No	985 (83.6%)	289 (82.1%)	696 (84.3%)	
Yes	193 (16.4%)	63 (17.9%)	130 (15.7%)	
Smoking history				0.762
No	939 (79.7%)	283 (80.4%)	656 (79.4%)	
Yes	239 (20.3%)	69 (19.6%)	170 (20.6%)	
History of alcohol consumption				0.364
No	847 (71.9%)	260 (73.9%)	587 (71.1%)	
Yes	331 (28.1%)	92 (26.1%)	239 (28.9%)	
Cardiovascular disease				0.927
No	1115 (94.7%)	334 (94.9%)	781 (94.6%)	
Yes	63 (5.35%)	18 (5.11%)	45 (5.45%)	
Hypertension				0.777
No	980 (83.2%)	295 (83.8%)	685 (82.9%)	
Yes	198 (16.8%)	57 (16.2%)	141 (17.1%)	
Cerebrovascular disorders				0.519
No	1142 (96.9%)	339 (96.3%)	803 (97.2%)	
Yes	36 (3.06%)	13 (3.69%)	23 (2.78%)	
Gastrointestinal bleeding				1.000
No	1154 (98.0%)	345 (98.0%)	809 (97.9%)	
Yes	24 (2.04%)	7 (1.99%)	17 (2.06%)	
Spontaneous bacterial peritonitis				0.769
No	1092 (92.7%)	328 (93.2%)	764 (92.5%)	
Yes	86 (7.30%)	24 (6.82%)	62 (7.51%)	
Biliary tract disorders				0.811
No	964 (81.8%)	290 (82.4%)	674 (81.6%)	
Yes	214 (18.2%)	62 (17.6%)	152 (18.4%)	
Co-infection				0.230
No	768 (65.2%)	220 (62.5%)	548 (66.3%)	
Yes	410 (34.8%)	132 (37.5%)	278 (33.7%)	
Hepatorenal syndrome				0.527
No	1166 (99.0%)	350 (99.4%)	816 (98.8%)	
Yes	12 (1.02%)	2 (0.57%)	10 (1.21%)	
Ascites				0.094
No	983 (83.4%)	304 (86.4%)	679 (82.2%)	
Yes	195 (16.6%)	48 (13.6%)	147 (17.8%)	
Hepatitis B				0.797
No	193 (16.4%)	56 (15.8%)	137 (16.6%)	
Yes	985 (83.6%)	298 (84.2%)	687 (83.4%)	
Hepatitis C				0.494
No	1127 (95.7%)	336 (94.9%)	791 (96.0%)	
Yes	51 (4.33%)	18 (5.08%)	33 (4.00%)	
Age (years)				0.499
< 55 years old	585 (49.7%)	169 (48.0%)	416 (50.4%)	
≥ 55 years old	593 (50.3%)	183 (52.0%)	410 (49.6%)	
Sex				0.308
Male	849 (72.1%)	246 (69.9%)	603 (73.0%)	
Female	329 (27.9%)	106 (30.1%)	223 (27.0%)	
GLU	5.03[4.50;5.97]	5.00[4.53;5.77]	5.04[4.49;6.03]	0.550
WBC	5.10[3.80;6.50]	4.90[3.70;6.12]	5.20[3.80;6.60]	0.136
NEUT	2.81[2.10;3.80]	2.70[2.00;3.70]	2.90[2.10;3.90]	0.023
LYT	0.14[0.08;0.25]	0.14[0.08;0.25]	0.14[0.08;0.25]	0.921
NEUP	58.5 (11.9)	57.0 (12.1)	59.1 (11.9)	0.007
LYP	2.90[1.60;4.90]	3.15[1.63;4.80]	2.87[1.56;4.90]	0.394
HGB	126 [101;145]	126 [99.0;147]	126 [102;144]	0.765
MCV	92.1[85.3;97.0]	92.0[85.5;96.0]	92.2[85.3;97.3]	0.609
MCH	30.7[27.8;32.4]	30.6[27.9;32.0]	30.7[27.7;32.6]	0.368
PLT	126 [72.0;186]	130 [76.0;185]	125 [70.0;186]	0.382
MPV	9.90[9.10;10.6]	9.80[9.00;10.6]	9.90[9.20;10.7]	0.106
RBC	4.26[3.45;4.88]	4.22[3.35;4.88]	4.29 [3.46;4.89]	0.542
PCT	0.12 [0.07;0.18]	0.12 [0.08;0.18]	0.12 [0.07;0.18]	0.540
K	3.80 [3.54;4.10]	3.80 [3.57;4.10]	3.80 [3.53;4.10]	0.968
Na	140 [138;142]	140 [138;142]	140 [137;142]	0.286
Cl	104 [102;106]	104 [102;107]	104 [101;106]	0.339
P	1.01 [0.89;1.13]	1.01 [0.88;1.14]	1.01 [0.89;1.13]	0.647
Mg	0.83 [0.76;0.90]	0.84 [0.76;0.90]	0.83 [0.76;0.90]	0.482
Ca	2.18 [2.06;2.29]	2.19 [2.05;2.28]	2.18 [2.06;2.29]	0.920
TG	0.94 [0.70;1.36]	0.94 [0.70;1.36]	0.95 [0.70;1.36]	0.873
CHOL	4.09 [3.30;4.98]	4.09 [3.29;5.06]	4.10 [3.30;4.97]	0.930
HDL	1.17 [0.92;1.43]	1.17 [0.90;1.43]	1.17 [0.94;1.43]	0.497
APOA1	1.24 [0.98;1.49]	1.23 [0.96;1.49]	1.25 [0.99;1.50]	0.615
APOB	0.78 [0.58;0.97]	0.78 [0.58;0.97]	0.77 [0.58;0.97]	0.794
VLDL	0.43 [0.32;0.62]	0.43 [0.32;0.61]	0.43 [0.32;0.62]	0.845
LDL	2.33 [1.72;3.09]	2.36 [1.75;3.09]	2.30 [1.69;3.08]	0.506
LDH	188 [158;235]	187 [157;228]	189 [158;237]	0.626
AHBDH	150 [125;188]	145 [125;187]	151 [126;188]	0.506
IgA	2.67 [1.91;3.36]	2.67 [1.88;3.32]	2.66 [1.92;3.38]	0.647
IgG	15.6 [12.8;19.5]	15.6 [12.5;19.5]	15.7 [12.9;19.5]	0.464
IgM	1.22 [0.81;1.78]	1.13 [0.76;1.68]	1.26 [0.85;1.84]	0.001
PT	14.4 [13.2;16.6]	14.3 [13.1;16.7]	14.4 [13.3;16.5]	0.509
PTA	83.0 [64.0;99.0]	83.5 [63.0;100]	82.0 [64.0;98.0]	0.508
INR	1.12 [1.01;1.35]	1.12 [1.00;1.36]	1.13 [1.01;1.34]	0.472
APTT	41.0 [37.4;45.7]	40.9 [37.6;45.5]	41.0 [37.3;45.8]	0.875
TT	17.5 [16.6;18.7]	17.6 [16.6;18.8]	17.5 [16.5;18.7]	0.524
D-Dimer	0.55 [0.25;2.14]	0.62 [0.24;2.38]	0.48 [0.25;2.01]	0.300
TP	66.7 [61.5;71.8]	66.6 [61.5;71.2]	66.8 [61.5;71.9]	0.571
ALB	37.9 [31.8;43.0]	38.0 [31.7;42.9]	37.9 [31.8;43.0]	0.949
GLO	28.2 [24.6;32.9]	28.4 [24.6;32.2]	28.2 [24.5;33.2]	0.674
AVSG	1.38 [1.02;1.69]	1.40 [1.06;1.70]	1.37 [1.02;1.68]	0.579
PA	156 [79.0;223]	156 [81.8;225]	155 [79.0;222]	0.746
CHE	5768 [3425;8271]	5646 [3485;8083]	5865 [3399;8273]	0.881
TBIL	20.3 [13.5;35.1]	20.0 [13.4;35.2]	20.4 [13.6;35.1]	0.521
DBIL	6.60 [4.10;14.5]	6.50 [4.07;14.1]	6.70 [4.10;14.6]	0.588
IBIL	13.4 [9.10;20.3]	13.3 [8.88;19.8]	13.5 [9.20;20.4]	0.444
TBA	18.4 [5.70;55.6]	18.3 [6.20;56.8]	18.4 [5.40;54.7]	0.768
AKP	83.0 [65.0;117]	82.0 [63.0;111]	83.0 [65.2;118]	0.266
GGT	37.0 [23.0;75.8]	37.0 [22.0;75.2]	38.0 [23.0;76.5]	0.419
ALT	29.0 [19.0;44.0]	29.0 [19.0;43.0]	29.0 [20.0;44.0]	0.472
AST	35.0 [27.0;52.8]	35.0 [26.0;50.0]	35.0 [27.0;54.0]	0.428
ADA	19.0 [14.0;27.2]	19.0 [13.0;27.0]	19.0 [14.0;27.8]	0.300
AFU	28.0 [23.0;36.0]	28.0 [21.1;35.0]	29.0 [23.0;36.0]	0.037
FIB	2.55 [1.96;3.14]	2.55 [1.95;3.16]	2.55 [1.96;3.13]	0.938
BUN	4.61 [3.70;5.78]	4.65 [3.80;5.97]	4.60 [3.69;5.70]	0.296
CREA	72.0 [61.0;84.0]	72.0 [59.0;87.2]	72.0 [62.0;83.0]	0.593
UA	309 [245;380]	306 [240;378]	311 [247;381]	0.432
CysC	0.88 [0.72;1.12]	0.88 [0.71;1.11]	0.89 [0.72;1.13]	0.549
CO2	24.2 [22.3;26.1]	24.4 [22.4;26.0]	24.2 [22.1;26.1]	0.658
SOD	151 [126;174]	150 [123;175]	151 [127;174]	0.453
CK	107 [70.0;165]	106 [69.0;153]	108 [70.2;172]	0.353
CRP	10.0 [4.50;22.5]	10.0 [4.48;23.6]	10.1 [4.50;22.1]	0.532
Ammo	40.1 [23.4;57.0]	40.0 [23.7;57.8]	40.1 [23.4;56.4]	0.820

### Comparison of hepatic encephalopathy and non-hepatic encephalopathy grouping

As shown in Table 3, among the 1178 included patients, 203 had hepatic encephalopathy and 975 did not have hepatic encephalopathy within six months of following up. The morbidity rate was 17.23%. The differences of the following variables between the hepatic encephalopathy and non-hepatic encephalopathy groups were statistically significant (*P* < 0.05) : age, diabetes mellitus (DM), ascites, gastrointestinal bleeding, spontaneous bacterial peritonitis, blood potassium (K), alkaline transaminase (AKP), glutamic aminotransferase (AST), and glutamic alanine transaminase (ALT) .

**Table 3:** Analysis of differences between the hepatic encephalopathy and non-hepatic encephalopathy groups.

Variables	Total (N = 1178)	Non-HE (N = 975)	HE (N = 203)	OR	*P* value
Gender	0.27 (0.44)	0.28 (0.45)	0.23 (0.43)	0.80 [0.52;1.25]	0.324
Age (years)	0.50 (0.50)	0.46 (0.50)	0.69 (0.47)	2.57 [1.72;3.84]	< 0.001
Smoking history	0.21 (0.40)	0.20 (0.40)	0.23 (0.43)	1.22 [0.78;1.91]	0.385
History of alcohol consumption	0.29 (0.45)	0.28 (0.45)	0.34 (0.47)	1.30 [0.87;1.94]	0.207
Cerebrovascular disease	0.03 (0.16)	0.03 (0.17)	0.02 (0.15)	0.81 [0.24;2.78]	0.742
Cardiovascular disease	0.05 (0.23)	0.05 (0.22)	0.06 (0.24)	1.19 [0.54;2.62]	0.664
Diabetes mellitus	0.16 (0.36)	0.14 (0.34)	0.27 (0.45)	2.39 [1.53;3.73]	< 0.001
Hypertension	0.17 (0.38)	0.16 (0.37)	0.20 (0.40)	1.29 [0.80;2.08]	0.29
Ascites	0.18 (0.38)	0.15 (0.36)	0.34 (0.47)	2.89 [1.89;4.41]	< 0.001
Gastrointestinal bleeding	0.02 (0.14)	0.02 (0.12)	0.05 (0.21)	3.07 [1.12;8.46]	0.03
Co-infection	0.34 (0.47)	0.33 (0.47)	0.38 (0.49)	1.22 [0.83;1.81]	0.317
Biliary tract disorders	0.18 (0.39)	0.18 (0.38)	0.23 (0.42)	1.37 [0.87;2.16]	0.178
Spontaneous bacterial peritonitis	0.08 (0.26)	0.05 (0.21)	0.23 (0.42)	5.90 [3.43;10.1]	< 0.001
Hepatorenal syndrome	0.01 (0.11)	0.01 (0.10)	0.02 (0.15)	2.37 [0.60;9.28]	0.216
Hepatitis B	0.83 (0.37)	1.00 (0.00)	0.04 (0.20)	1.5 [0.78;3.53]	0.987
Hepatitis C	0.04 (0.20)	0.04 (0.18)	0.06 (0.24)	1.84 [0.84;4.04]	0.130
WBC	5.47 (2.55)	5.51 (2.64)	5.27 (2.00)	0.96 [0.89;1.04]	0.341
NEUT	3.33 (2.15)	3.36 (2.24)	3.16 (1.57)	0.95 [0.86;1.05]	0.341
NEUP	59.1 (11.9)	59.1 (11.9)	59.0 (11.5)	1.00 [0.98;1.01]	0.895
LYT	0.19 (0.20)	0.19 (0.20)	0.19 (0.16)	0.90 [0.34;2.41]	0.836
LYP	3.77 (3.45)	3.76 (3.51)	3.83 (3.11)	1.01 [0.95;1.06]	0.817
RBC	4.16 (1.02)	4.18 (1.02)	4.06 (1.03)	0.89 [0.74;1.07]	0.225
HGB	122 (29.0)	122 (29.1)	121 (28.3)	1.00 [0.99;1.01]	0.725
MCV	90.1 (11.9)	89.8 (11.9)	91.7 (11.5)	1.01 [1.00;1.03]	0.092
MCH	29.6 (4.60)	29.5 (4.63)	30.2 (4.41)	1.04 [0.99;1.08]	0.106
PLT (× 10^9^/L)	135 (82.3)	135 (82.4)	131 (81.4)	1.00 [1.00;1.00]	0.604
MPV	9.97 (1.32)	9.96 (1.28)	10.0 (1.52)	1.05 [0.91;1.21]	0.539
PCT	0.13 (0.08)	0.13 (0.08)	0.13 (0.08)	1.04 [0.09;11.5]	0.975
K	3.83 (0.50)	3.84 (0.50)	3.74 (0.49)	0.65 [0.45;0.96]	0.028
Na	139 (4.04)	139 (4.10)	139 (3.67)	1.02 [0.97;1.07]	0.544
Cl	104 (4.33)	104 (4.32)	104 (4.41)	1.01 [0.97;1.06]	0.672
P	1.01 (0.21)	1.02 (0.21)	1.00 (0.20)	0.70 [0.28;1.74]	0.439
Mg	0.82 (0.11)	0.82 (0.11)	0.82 (0.12)	1.24 [0.23;6.70]	0.806
Ca	2.17 (0.17)	2.17 (0.17)	2.15 (0.18)	0.52 [0.18;1.53]	0.233
TG (mmol/L)	1.17 (0.95)	1.16 (0.95)	1.19 (0.95)	1.03 [0.86;1.24]	0.714
HDL	1.18 (0.41)	1.19 (0.41)	1.14 (0.42)	0.77 [0.49;1.22]	0.264
APOA1	1.22 (0.40)	1.23 (0.40)	1.17 (0.42)	0.67 [0.42;1.06]	0.089
APOB	0.79 (0.30)	0.79 (0.31)	0.80 (0.29)	1.06 [0.57;1.95]	0.864
LDL	2.44 (1.06)	2.45 (1.07)	2.40 (0.98)	0.95 [0.80;1.14]	0.612
VLDL	0.53 (0.43)	0.53 (0.43)	0.55 (0.44)	1.12 [0.76;1.64]	0.563
LDH	205 (78.3)	203 (68.7)	217 (117)	1.00 [1.00;1.00]	0.072
CK	139 (120)	142 (125)	127 (90.1)	1.00 [1.00;1.00]	0.189
IgA	2.79 (1.29)	2.78 (1.25)	2.84 (1.50)	1.04 [0.90;1.19]	0.639
IgG	16.6 (5.50)	16.6 (5.46)	16.6 (5.72)	1.00 [0.97;1.03]	0.975
IgM	1.47 (0.96)	1.45 (0.90)	1.56 (1.20)	1.11 [0.93;1.33]	0.262
PT	15.3 (2.97)	15.3 (2.97)	15.3 (3.03)	1.01 [0.95;1.07]	0.793
PTA	81.2 (22.4)	81.2 (22.2)	81.0 (23.4)	1.00 [0.99;1.01]	0.928
AHBDH	164 (63.1)	162 (55.9)	172 (92.7)	1.00 [1.00;1.00]	0.125
INR	1.22 (0.32)	1.22 (0.31)	1.23 (0.32)	1.12 [0.63;2.00]	0.708
APTT	42.3 (7.65)	42.2 (7.58)	43.2 (8.00)	1.02 [0.99;1.04]	0.156
TT	17.8 (2.11)	17.9 (2.10)	17.7 (2.15)	0.96 [0.87;1.06]	0.388
FIB	2.63 (0.93)	2.62 (0.92)	2.71 (0.99)	1.10 [0.91;1.34]	0.317
D-Dimer	1.77 (3.02)	1.81 (3.13)	1.57 (2.34)	0.97 [0.90;1.04]	0.411
ALB	37.1 (7.39)	37.1 (7.35)	36.6 (7.63)	0.99 [0.96;1.01]	0.403
TP	66.4 (8.07)	66.5 (8.13)	66.0 (7.76)	0.99 [0.97;1.02]	0.506
GLO	29.4 (7.19)	29.4 (7.22)	29.4 (7.06)	1.00 [0.98;1.03]	0.908
AVSG	1.35 (0.45)	1.35 (0.44)	1.33 (0.46)	0.89 [0.58;1.35]	0.578
CHE	5976 (2934)	6006 (2940)	5813(2907)	1.00 [1.00;1.00]	0.493
TBIL	34.5 (50.5)	33.4 (50.0)	40.1 (52.8)	1.00 [1.00;1.01]	0.18
DBIL	16.5 (35.3)	15.7 (34.7)	20.9 (38.2)	1.00 [1.00;1.01]	0.138
TBA	44.2 (63.9)	43.1 (63.9)	50.3 (63.9)	1.00 [1.00;1.00]	0.243
IBIL	18.2 (17.6)	17.8 (17.6)	19.9 (17.6)	1.01 [1.00;1.02]	0.219
AKP(μ/L)	102 (68.4)	99.2 (63.2)	116 (90.9)	1.00 [1.00;1.01]	0.018
GGT	79.6 (173)	78.0 (176)	88.3 (152)	1.00 [1.00;1.00]	0.544
AST(μ/L)	52.8 (78.4)	49.6 (62.1)	70.2 (136)	1.00 [1.00;1.00]	0.025
ADA	21.6 (10.5)	21.3 (10.3)	23.1 (11.7)	1.02 [1.00;1.03]	0.075
AFU	30.2 (10.5)	30.0 (10.5)	31.1 (10.5)	1.01 [0.99;1.03]	0.27
CREA(umol/L)	75.4 (26.4)	75.1 (24.8)	77.1 (34.0)	1.00 [1.00;1.01]	0.449
BUN	5.20 (2.89)	5.25 (3.00)	4.94 (2.17)	0.96 [0.89;1.03]	0.273
UA	319 (107)	320 (109)	316 (97.6)	1.00 [1.00;1.00]	0.682
CysC	1.02 (0.78)	1.02 (0.78)	1.06 (0.80)	1.06 [0.86;1.30]	0.582
CO2	24.1 (3.22)	24.0 (3.19)	24.1 (3.36)	1.01 [0.95;1.07]	0.771
SOD	148 (35.4)	148 (35.1)	147 (37.2)	1.00 [0.99;1.00]	0.559
CRP	16.2 (19.7)	16.0 (18.5)	17.5 (25.3)	1.00 [0.99;1.01]	0.442
Ammo	40.9 (22.4)	40.5 (22.5)	42.7 (22.2)	1.00 [1.00;1.01]	0.325
PA	155 (82.3)	155 (81.3)	150 (87.5)	1.00 [1.00;1.00]	0.491
ALT(μ/L)	46.6 (109)	40.5 (42.5)	79.7 (257)	1.00 [1.00;1.01]	0.004
CHOL	4.18 (1.34)	4.20 (1.36)	4.11 (1.22)	0.95 [0.82;1.10]	0.479
GLU	5.80 (2.70)	5.84 (2.74)	5.63 (2.46)	0.97 [0.90;1.05]	0.426

### Assignment of variables in the model for risk prediction of hepatic encephalopathy

A total of 1 dependent variable (occurrence of hepatic encephalopathy) and 77 independent variables were included in this study. Table 4 assigned values to the variables individually and converted the corresponding variables to categorical variables, including the assignment of dichotomous variables and the handling of dummy variables of multicategorical variables. The occurrence of HE was set as the dependent variable Y. The independent continuous variables (X) such as white blood cell count, platelet count, hemoglobin, glutamate aminotransferase, blood creatinine, blood urea nitrogen, urea, glucose, total cholesterol, triglycerides, total bile acids, albumin, were still included in the model analysis as numerical variables.

**Table 4:** Variable assignment.

Variable name	Variable name	Variable value representation and meaning
Y	Whether hepatic encephalopathy occurs	No = 0, Yes = 1
X1	Sex	Male = 0, Female = 1
X2	Age (years)	< 55 years old = 0, ≥ 55 years old = 1
X3	Marital status	X3-1 = 0:unmarried, X3-2 = 1:married, X3-3 = 2:other
X4	Smoking history	X4 = 1: Yes; X4 = 0: No
X5	History of alcohol consumption	X5 = 1: Yes; X5 = 0: No
X6	Cerebrovascular disease	X6 = 1: Yes; X6 = 0:No
X7	Cardiovascular disease	X7 = 1: Yes; X7 = 0: No
X8	Diabetes	X8 = 1: Yes; X8 = 0: No
X9	History of hypertension	X9 = 1: Yes; X9 = 0: No
X10	Ascites	X10 = 1: Yes; X10 = 0: No
X11	Gastrointestinal bleeding	X11 = 1: Yes; X11 = 0: No
X12	Infection	X12 = 1: Yes; X12 = 0: No
X13	Biliary tract disorders	X13 = 1: Yes; X13 = 0: No
X14	Spontaneous bacterial peritonitis	X14 = 1: Yes; X14 = 0: No
X15	Hepatorenal syndrome	X15 = 1: Yes; X15 = 0: No
X16	Hepatitis B	X16 = 1: Yes; X16 = 0: No
X17	Hepatitis C	X17 = 1: Yes; X17 = 0: No
X18	WBC	Numeric variables
X19	NEUT	Numeric variables
X20	LYT	Numeric variables
X21	LYP	Numeric variables
X22	RBC	Numeric variables
X23	HGB	Numerical variables
X24	MCV	Numerical variables
X25	MCH	Numerical variables
X26	PLT	Numerical variables
X27	MPV	Numerical variables
X28	PCT	Numerical variables
X29	K	Numerical variables
X30	Na	Numerical variables
X31	Cl	Numerical variables
X32	P	Numerical variables
X33	Mg	Numerical variables
X34	Ca	Numerical variables
X35	TG	Numerical variables
X36	HDL	Numerical variables
X37	APOA1	Numerical variables
X38	APOB	Numerical variables
X39	LDL	Numerical variables
X40	VLDL	Numerical variables
X41	LDH	Numerical variables
X42	CK	Numerical variables
X43	IgA	Numerical variables
X44	IgG	Numerical variables
X45	IgM	Numerical variables
X46	PT	Numerical variables
X47	PTA	Numerical variables
X48	AHBDH	Numerical variables
X49	INR	Numerical variables
X50	APTT	Numerical variables
X51	TT	Numerical variables
X52	FIB	Numerical variables
X53	D-Dimer	Numerical variables
X54	ALB	Numerical variables
X55	TP	Numerical variables
X56	GLO	Numerical variables
X57	AVSG	Numerical variables
X58	CHE	Numerical variables
X59	TBIL	Numerical variables
X60	DBIL	Numerical variables
X61	TBA	Numerical variables
X62	IBIL	Numerical variables
X63	AKP	Numerical variables
X64	GGT	Numerical variables
X65	AST	Numerical variables
X66	ADA	Numerical variables
X67	AFU	Numerical variables
X68	CREA	Numerical variables
X69	BUN	Numerical variables
X70	UA	Numerical variables
X71	CysC	Numerical variables
X72	CO2	Numerical variables
X73	SOD	Numerical variables
X74	CRP	Numerical variables
X75	Ammo	Numerical variables
X76	PA	Numerical variables
X77	ALT	Numerical variables
X78	CHOL	Numerical variables
X79	GLU	Numerical variables

### Univariate logistic regression analysis

The occurrence of hepatic encephalopathy was used as the dependent variable Y, and all candidate predictors based on 826 patients in the training set were used as independent variables. Univariate logistic regression analysis was performed to screen the potential predictors. The results shown that the following variables were considered to be the potential predictors and were entered into the regression equation (Table 5): age, serum alkaline transaminase (AKP), glutamic aminotransferase (ALT), adenosine deaminase (ADA), glutamic aminotransferase (AST), diabetes mellitus, serum lactate dehydrogenase (LDH), apolipoprotein A, serum potassium, red blood cell volume (MCV), ascites, spontaneous bacterial peritonitis, and gastrointestinal bleeding.

**Table 5:** Results of univariate logistic regression analysis.

Variables	*Β*	SD	OR	95% CI	Z	*P*
Age	0.943	0.20524	2.569	1.718–3.841	4.597	0
ADA	0.015	0.00861	1.015	0.998–1.033	1.781	0.075
AST	0.002	0.00103	1.002	1–1.004	2.234	0.025
ALT	0.004	0.00141	1.004	1.001–1.007	2.865	0.004
AKP	0.003	0.00118	1.003	1–1.005	2.367	0.018
History of diabetes mellitus	0.871	0.22696	2.389	1.531–3.727	3.837	0
Serum lactate dehydrogenase	0.002	0.00108	1.002	1–1.004	1.801	0.072
Apolipoprotein A	− 0.405	0.23813	0.667	0.418–1.064	− 1.7	0.089
Serum potassium	− 0.428	0.19476	0.652	0.445–0.955	− 2.196	0.028
Red blood cell volume	0.014	0.00847	1.014	0.998–1.031	1.685	0.092
Ascites	1.061	0.21522	2.889	1.895–4.406	4.93	0
Spontaneous bacterial peritonitis	1.775	0.27639	5.903	3.434–10.147	6.424	0
Gastrointestinal bleeding	1.122	0.51694	3.072	1.115–8.46	2.171	0.03

### Predictors screening

**Figure 2:**
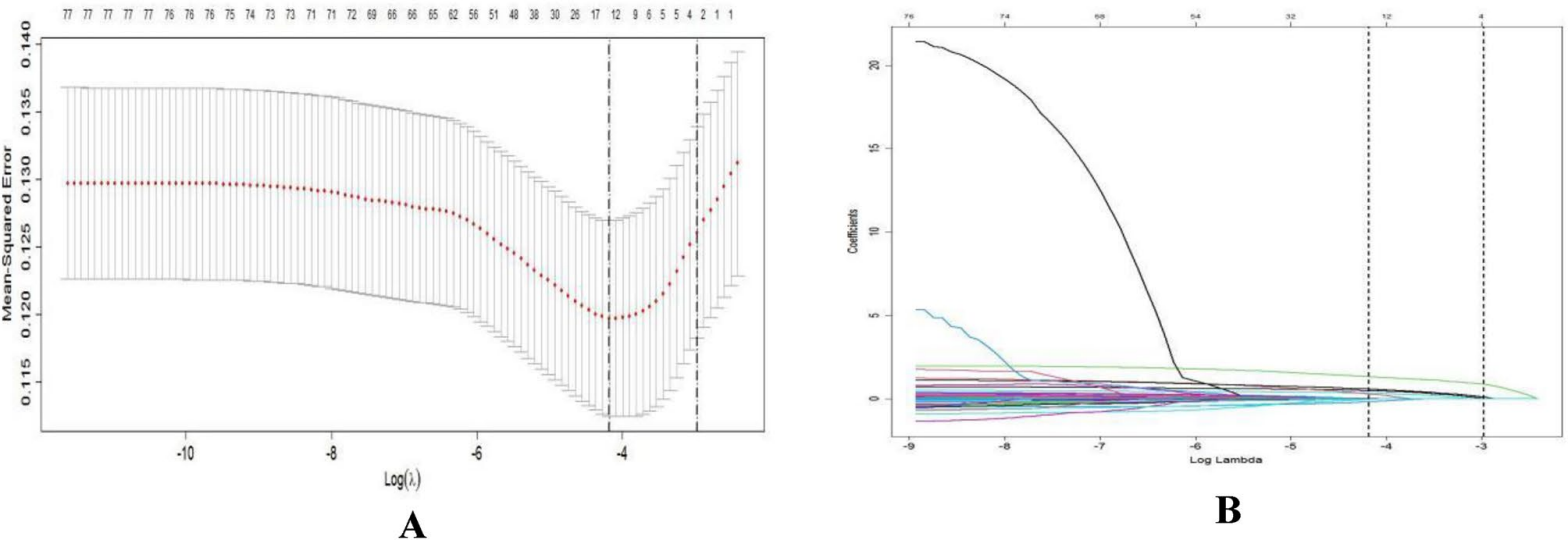
LASSO regression diagram.

LASSO regression was performed using the "glmnet" package in R software. All independent variables were screened using LASSO regression, and the adjustment parameter λ was validated using the ten-fold crossover method. Conversely, if the regression coefficient is not zero, it indicates that the variable is strongly associated with the occurrence of HE in patients with cirrhosis. The two dashed lines indicate lambda.min, which represents the value of λ corresponding to the smallest error and which can correspond to the least number of predictor variables, and lambda.1se, which represents the value of λ for the most streamlined model within one standard error of lambda.min. All the independent variables in this study were screened by LASSO regression, and finally 15 variables with non-zero regression coefficients were output at lambda.min. These 15 variables were listed below: age, sex, diabetes mellitus (DM), spontaneous bacterial peritonitis, ascites, gastrointestinal bleeding, serum α-hydroxybutyrate dehydrogenase (α-HBDH), white blood cells ( WBC), red blood cell volume (MCV), serum potassium (K), prothrombin time (TT), serum alkaline phosphatase (AKP), alanine transaminase (ALT), adenosine deaminase (ADA), and plasma ammonia (Ammo). The above 15 predictor variables were included in a multifactorial logistic regression analysis, and six fo these 15 variables showed statistically significant differences (*P* < 0.05) (Fig. 2A, B, Table 6).

**Table 6:** Multifactorial logistic regression analysis based on LASSO regression.

Variables	*β*	SD	OR	95% CI	Z	*P*
Age	0.815	0.22239	2.26	1.461–3.494	3.666	0
History of diabetes mellitus (DM)	0.504	0.25423	1.656	1.006–2.725	1.983	0.047
Ascites	0.706	0.24217	2.025	1.26–3.255	2.914	0.004
Spontaneous bacterial peritonitis (SBP)	1.58	0.30707	4.856	2.66–8.865	5.146	0
ALT, U/L	0.005	0.00142	1.005	1.002–1.007	3.216	0.001
K, mmol/L	− 0.414	0.2065	0.661	0.441–0.99	− 2.007	0.045

### Establishment and demonstration of the model

**Figure 3:**
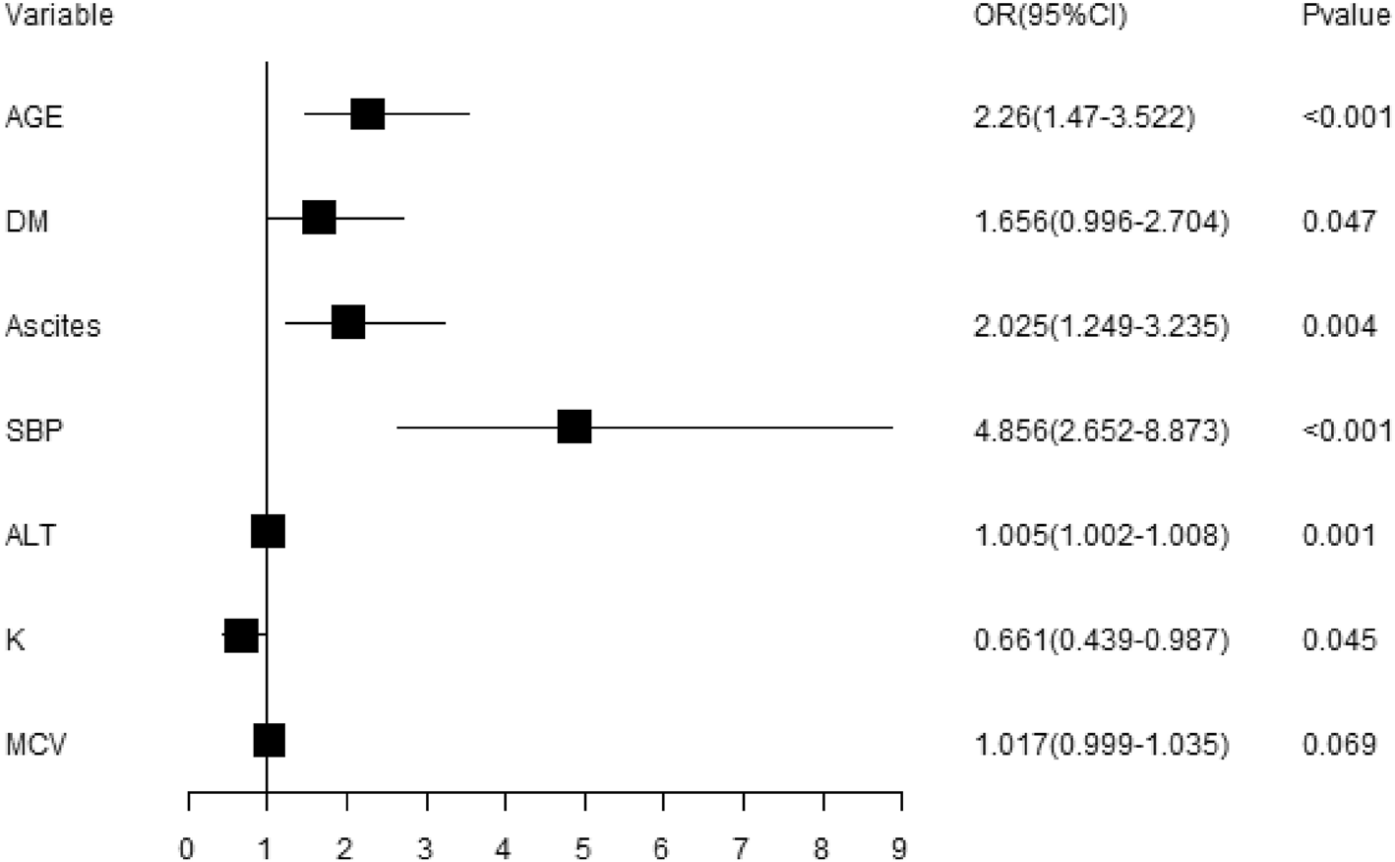
OR (95% CI) forest plot of predictors.

The risk prediction model for the occurrence of HE in patients with decompensated cirrhosis was established based on the above six predictors (risk factors), and the OR values obtained after incorporating the model are shown in Fig. 3, in which the combination of SBP or not had the greatest effect on the occurrence of hepatic encephalopathy, and the risk of getting hepatic encephalopathy in decompensated cirrhosis patients with SBP was 4.856 times higher than that in patients without SBP (2.66, 8.865); the risk of getting hepatic encephalopathy in decompensated cirrhosis patients older than 55 years of age was 2.26 times higher than in patients not older than 55 years (1.461, 3.494); the risk of getting hepatic encephalopathy in decompensated cirrhosis patients with a history of diabetes mellitus was 1.656 times higher than in patients without a history of diabetes mellitus (1.006, 2.725); the risk of getting hepatic encephalopathy in decompensated cirrhosis patients with ascites was 2.025 times higher than in patients without ascites (1.26, 3.255); the risk of getting hepatic encephalopathy increased incrementally with the increasing serum glutamate values in decompensated cirrhosis patients, with an OR value of 1.005 (1.002, 1.007).

**Figure 4:**
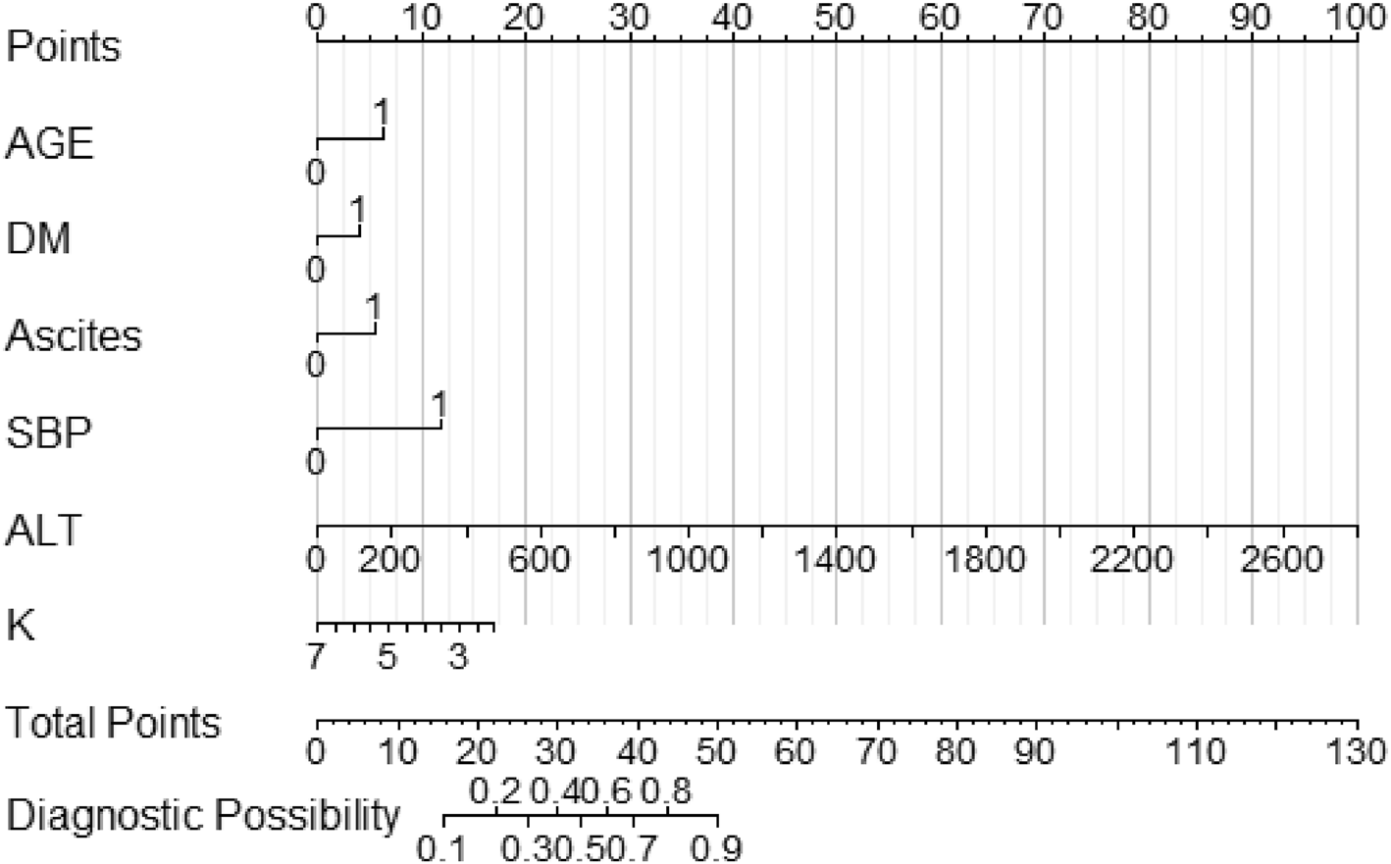
Nomogram for predicting the risk of getting hepatic encephalopathy in patients with decompensated cirrhosis. Note: AGE: age; DM: history of diabetes mellitus; SBP: spontaneous bacterial peritonitis; ALT: glutamic aminotransferase; K: serum potassium concentration; 1 = yes; 0 = no.

In order to visualize the weight of each predictor in the model and to visualize the model for clinical application, we used R software to construct a nomogram to demonstrate the model. The scores and risks of each predictor are shown in Fig. 4, Tables 7, and 8. The higher the total score, the higher the risk of developing hepatic encephalopathy.

**Table 7:** Total scores of the predictors and their corresponding probability of diagnosis of hepatic encephalopathy.

Total scores	16	22	26	30	33	36	39	44	50
Probability of diagnosis	0.1	0.2	0.3	0.4	0.5	0.6	0.7	0.8	0.9

**Table 8:** Scores for each predictor in the nomogram.

Predictor	AGE		DM		Ascites		SBP		ALT (U/L)		K (mmol/L)	
	A	S	A	S	A	S	A	S	A	S	A	S
	0	0	0	0	0	0	0	0	0	0	2.0	17
	1	6	1	4	1	6	1	12	200	7	2.5	15
									400	14	3.0	14
									600	21	3.5	12
									800	29	4.0	10
									1000	36	4.5	9
									1200	43	5.0	7

AGE: age; DM: history of diabetes mellitus; SBP: spontaneous bacterial peritonitis; ALT: glutamic aminotransferase; K: serum potassium concentration; A: assignment; S: score.

**Figure 5:**
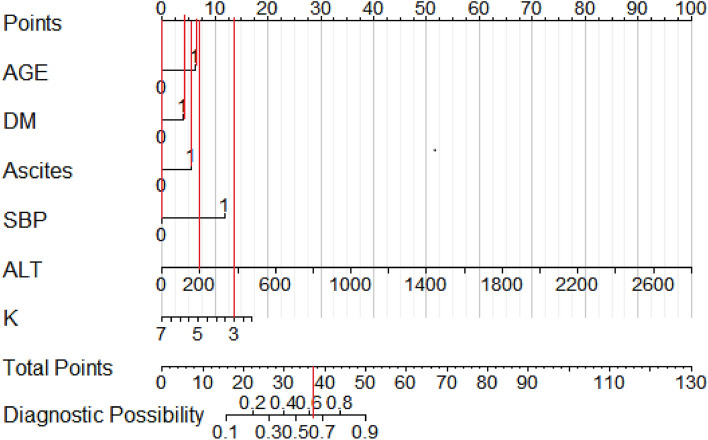
Example of the application of the established nomogram. Note: AGE: age; DM: history of diabetes mellitus; SBP: spontaneous bacterial peritonitis; ALT: glutamic aminotransferase; K: serum potassium concentration; 1 = yes; 0 = no.

The following example (see Fig. 5) illustrates the clinical application of the nomogram model for predicting the risk of getting hepatic encephalopathy: for example, a decompensated cirrhosis patients is 60 years old (6 points), he/she has diabetes mellitus (4 points), he is found to have ascites (6 points), he does not have spontaneous bacterial peritonitis (0 points), and laboratory tests suggested the value of his glutamic aminotransferase is 200 U/L (7 points), the value of his serum potassium concentration is 3.0 mmol /L (14 points). Then, this patient had a final score of 37, and his probability of developing hepatic encephalopathy would be greater than 0.6. That is to say, this patient had a high probability (risk) of developing hepatic encephalopathy, suggesting the need for timely and early intervention by medical personnel to reduce his risk of developing hepatic encephalopathy.

## Evaluation and validation of the model

### Distinguishability

**Figure 6:**
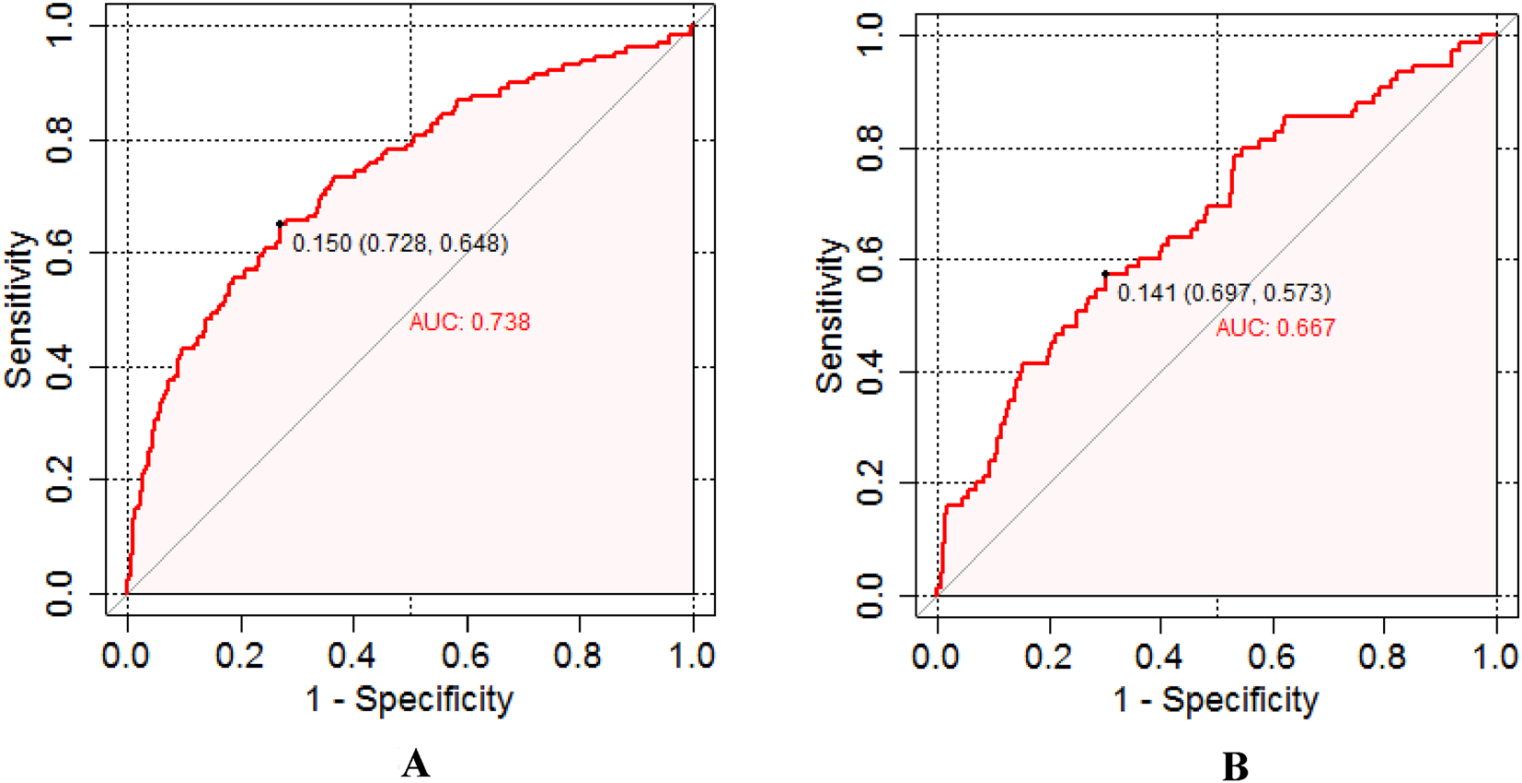
ROC curves of the training set (**A**) and validation set (**B**). Note: The area under the ROC curve for the model is 0.738 and 0.667 for the training and validation sets, respectively.

The distinguishability of the model was evaluated using ROC (receiver operator charteristics) curves (as shown in Fig. 6A, B), and the results showed that the AUC of the model was 0.738 (95% CI 0.63–0.746) in the training set (as shown in Fig. 6A) and 0.667 (95% CI 0.541–0.706) (as shown in Fig. 6B), and the AUCs of two sets indicated that the nomogram model was greatly differentiated. According to the Cut-Off value determined by the *Jorden index*, when the Cut-Off value of the training set was taken as 0.150, the sensitivity of the model was 72.8%, the specificity was 64.8%, the PPV was 30.4%, and the NPV was 91.9%; when the Cut-Off value of the validation set was taken as 0.141, the sensitivity of the model was 69.7%, the specificity was 57.3%, the PPV was 34.5%, and the NPV was 84.7%.

### Calibration

**Figure 7:**
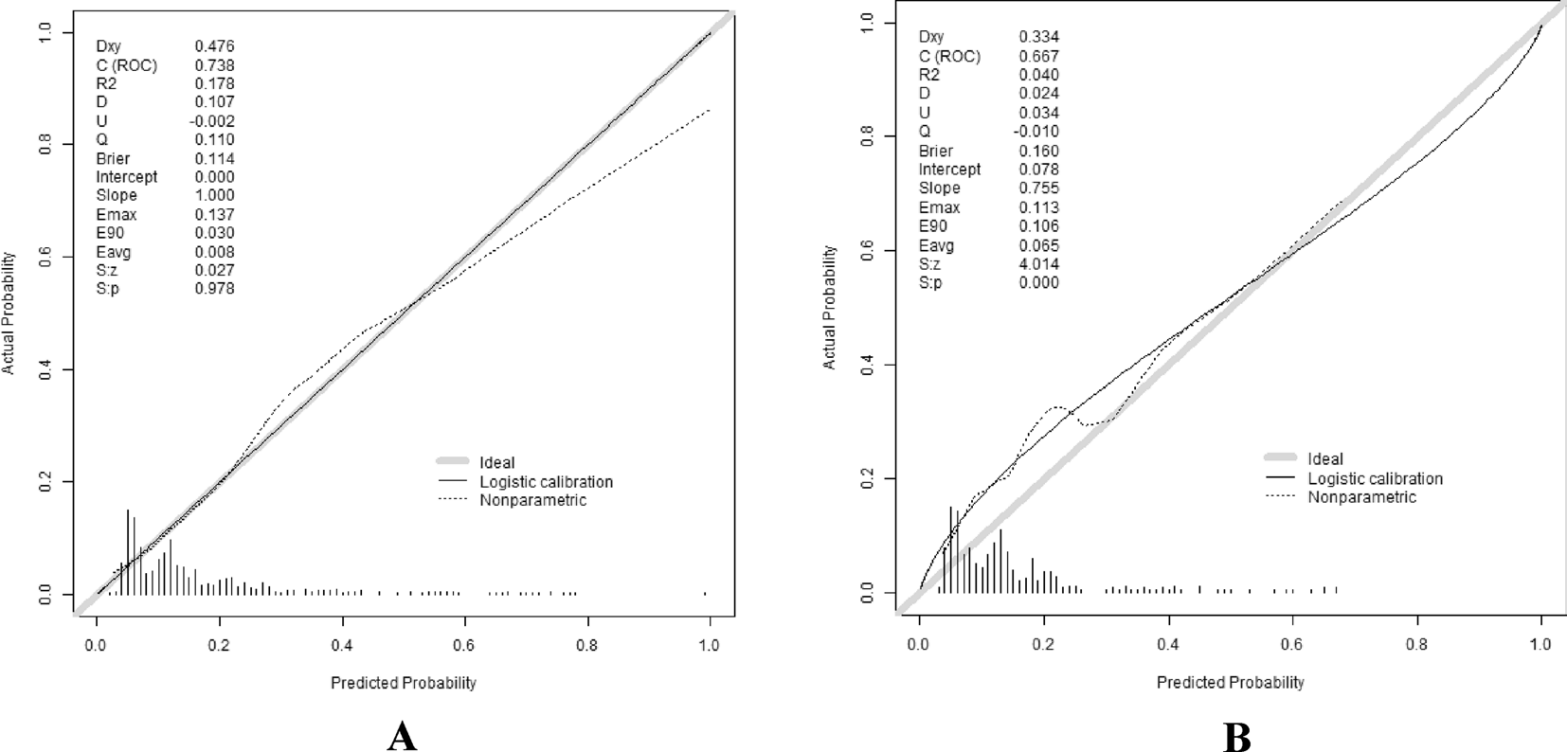
Calibration curves of the training set (**A**) and validation set (**B**). Note: The X-axis is the predicted probability of developing hepatic encephalopathy in patients with decompensated cirrhosis, and the Y-axis is the actual probability of developing hepatic encephalopathy in patients with decompensated cirrhosis. The diagonal dashed line indicates a perfect prediction, while the solid line indicates the actual corrected prediction.

Bootstrap sampling method was used to perform the calibration. Patients in the training and validation sets were repeatedly sampled 1000 times, respectively, and the calibration curves were plotted after validation. The horizontal coordinate indicates the likelihood of developing hepatic encephalopathy in patients with decompensated cirrhosis, and the vertical coordinate indicates the actual event occurrence. The further the calibration curve deviates from the diagonal, the greater the error (as shown in Fig. 7A, B). The Hosmer–Lemeshow test for goodness of fit was also applied, and the results showed that χ^2^ = 1.237587, *P* = 0.998 in the training set, χ2 = 31.90904, *P* = 0.0202 in the validation set, indicating that there was no significant difference between the predicted and actual observed values.

### Clinical decision curve analysis

**Figure 8:**
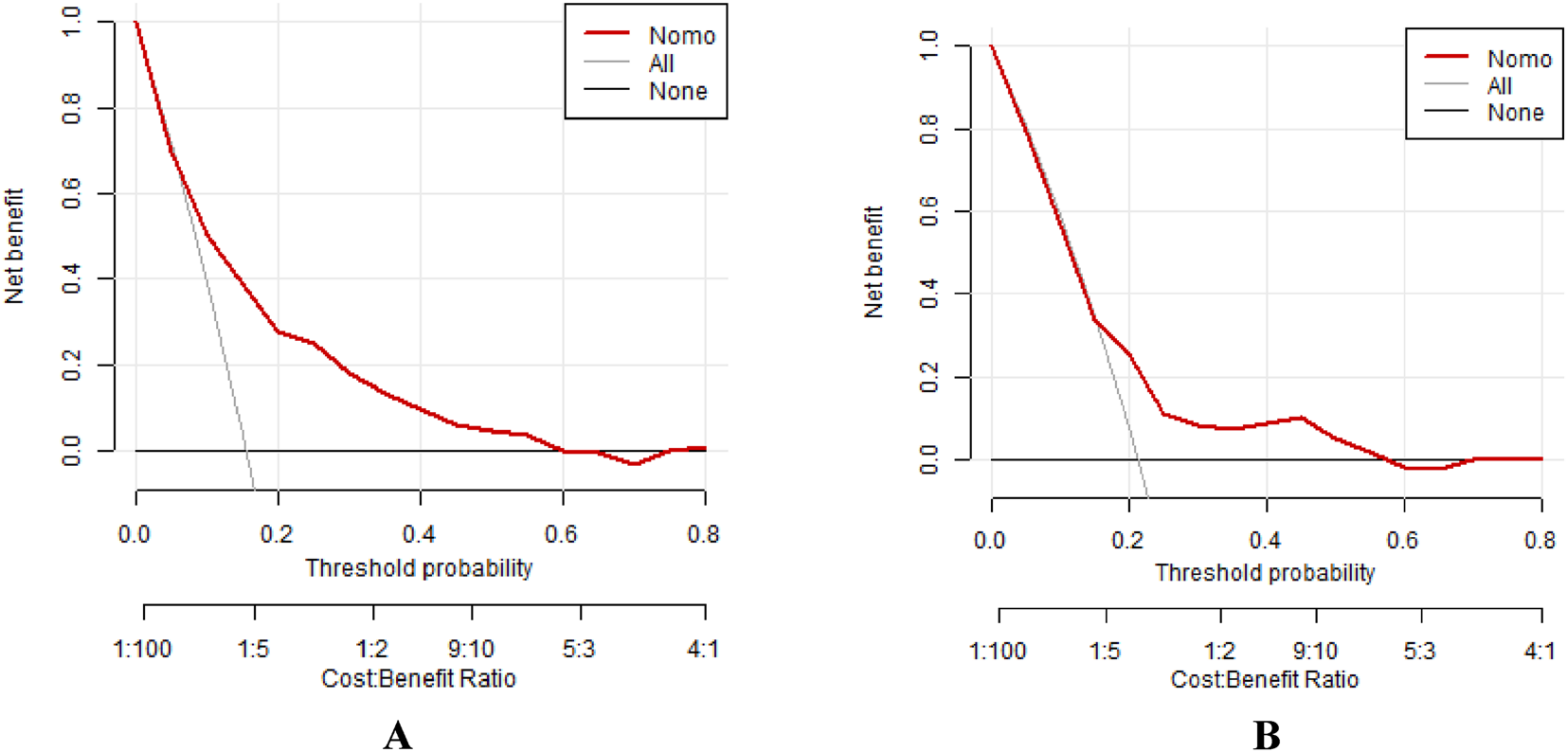
Calibration curves of the training set (**A**) and validation set (**B**). Note: The horizontal axis indicates that no patient received treatment after the application of the model, with a net benefit of 0. The diagonal line indicates that all patients received treatments.

We used clinical decision curve analysis (DCA) to assess the net benefit of the model in clinical application. As shown in (Fig. 8A, B), the results of the DCA show that the model has good clinical benefit in both the training and validation sets when compared to two extreme clinical scenarios (all patients received treatment or none of them received).

## Discussion

Hepatic encephalopathy is a complex disease with a wide range of etiologies and varying degrees of severity of morbidity. The use of appropriate measurement tools to assess the risk of getting hepatic encephalopathy can help to develop targeted interventions to reduce the occurrence of hepatic encephalopathy, which is important to improve patients’ quality of life and reduce the burden of medical care. Therefore, the development of high-quality risk prediction tools has become the focus of research on the prevention and treatment of hepatic encephalopathy. In recent years, scholars in various countries have constructed various risk prediction models for the development of hepatic encephalopathy based on the characteristics of the local population and epidemiological data. However, those risk prediction models constructed are diverse, the predictive indicators incorporated in each model are not consistent, the assessment contents and applicable population are not uniform, resulting in a certain gap between the prediction results and the real situation.

Risk prediction model studies aim to estimate the probability of an event occurring in an individual and can be divided into diagnostic models (presence or absence of a disease or symptom) and prognostic models (whether a specific outcome will occur in the future)^2^. The common metrics used to evaluate predictive models are the degree of discrimination and calibration, and if a good degree of discrimination is available, it indicates that the predictive model can accurately distinguish high-risk population with different risks. AUC value of 0.50 indicates that the model has predictive power but poor discrimination, 0.51–0.70 indicates that the model has low discrimination, 0.71–0.90 indicates good discrimination, and higher than 0.90 indicates high discrimination^3^. Sensitivity reflects the ability to correctly detect positive diagnosis patients, also known as the true positive rate, and specificity reflects the ability to correctly determine people who are actually disease-free as true negatives, known as the true negative rate^4^.

The risk prediction models can be divided into traditional statistical algorithm models and machine learning algorithm models according to the model building method. Traditional statistical algorithmic models are mathematical models based on statistical analysis of risk factors, i.e., the probability of disease occurrence is calculated by constructing mathematical models in which factors that can independently predict the occurrence of an event are selected as predictors. The most common models are logistic regression and Cox proportional risk regression models. Takikawa^5^ applied logistic regression analysis to construct a predictive model for the risk of developing hepatic encephalopathy, and the findings suggest that advanced age, prolonged prothrombin time, and high total serum bilirubin can be used as risk predictors for the development of hepatic encephalopathy. Although the specificity of this study was very high, its sensitivity was low, indicating that the inclusion of the above factors alone was not sufficient to predict the development of hepatic encephalopathy. In 2019, Labenz^6^ used history of minimal hepatic encephalopathy, history of hepatic encephalopathy, C-reactive protein, albumin, MELD score, serum interleukin 6 (IL-6) as predictors to establish a prediction model to validate the predictive value of IL-6 to identify the occurrence of hepatic encephalopathy, and the results showed that the predictive performance was substantially improved (AUC of 0.931).

In contrast to the logistic regression model, the Cox proportional risk regression model uses survival outcome and survival time as dependent variables, allowing simultaneous analysis of the effects of numerous factors on survival to study the incidence at different time points. Tapper^7^ used demographic, clinical, laboratory, and pharmacological data to construct a predictive model for the risk of developing hepatic encephalopathy based on the Cox proportional risk regression model, and The final prediction model consisted of four predictors: albumin, bilirubin, statin usage and non-selective β-blocker usage.The model was validated using bootstrapping and obtained an AUC of 0.73, indicated a hi gh degree of discrimination.

In this study, we chose to use logistic regression analysis to construct our analytical model, as opposed to opting for Cox's proportional risk model for several reasons: (1) the purpose of our study was to examine the impact of specific risk factors on a dichotomous outcome variable (whether or not a specific event occurs) rather than the impact on survival time. Therefore, we considered that logistic regression was more appropriate for our study, whereas the Cox proportional risk model was more appropriate for survival analysis. (2) Our dataset did not contain information on survival times, nor did we record the start and end times of observations for individuals, so it’s hard to perform analyses using the Cox proportional risk model. (3) Some of the independent variables in our dataset are categorical or ordinal, whereas the Cox's proportional risk model requires the independent variables to be continuous or dichotomous. If we convert these variables to dichotomous variables, we may lose some information and precision. Logistic regression, on the other hand, can handle multi-categorical or sequential variables and only requires dummy variable coding^8,9^. (4) There are some independent variables in our data set that may not meet the basic assumption of the Cox's proportional risk model, i.e., the assumption of equal proportional risk. This means that the impact of the independent variable on the outcome variable changes over time. If the equal proportional risk assumption does not hold, the results of the Cox's proportional risk model will lose their explanatory power. Logistic regression, on the other hand, does not require this assumption and is more flexible and robust.

In this study, we refered to the prevailing practice of the popular clinical prediction models^10–29^ and chose the logistic regression method to develop a nomogram model for predicting the risk of developing hepatic encephalopathy in patients with decompensated cirrhosis, and the model results were as described above. The results showed that the model performed well in terms of differentiation, calibration and clinical applicability and can be used in clinical practice.

The results obtained in this study suggested that age, diabetes mellitus (DM), ascites, spontaneous bacterial peritonitis (SBP), abnormal glutamate aminotransferase (ALT), and abnormal blood potassium (K) are risk factors (predictors) for the development of hepatic encephalopathy. These predictors were finally entered into the subsequent analysis and were used to build the nomogram model.

We included both potassium and sodium in the possible risk factors, and after statistical analysis, the difference in potassium was statistically significant (*P* < 0.05), while the difference in sodium was not statistically significant (*P* > 0.05), so we finally included potassium as an independent factor in the nomogram model for risk prediction of hepatic encephalopathy. It is worth noting that potassium is the major intracellular cation involved in maintaining electrolyte homeostasis and acid–base balance inside and outside the cell. Hypokalemia is defined as a serum potassium concentration less than 3.5mmol/L, which is commonly found in patients with liver cirrhosis, especially when combined with ascites, diuresis, vomiting, and diarrhea^30^. Hypokalemia may lead to metabolic alkalosis, which in turn promotes the occurrence of hepatic encephalopathy. Sodium, on the other hand, is the major extracellular cation involved in maintaining body fluid volume and osmolality. The impact of blood sodium abnormalities on hepatic encephalopathy is unclear. In light of this, we have also included a number of relevant studies in the Discussion section that support the rationale for choosing potassium rather than sodium as an independent factor in the development of hepatic encephalopathy in cirrhosis^30–34^.

In this study, the ROC curve was used to evaluate the predictive ability of the model, and the area under the ROC curve was calculated to evaluate the model performance. The accuracy of the model was evaluated by plotting the calibration curve. The clinical benefit of the model was evaluated using decision curve analysis (DCA). DCA is a method to evaluate prediction models by calculating the net clinical benefit. The results of the DCA showed that the risk prediction model established in this study had good clinical benefit in both the training set and the validation set when compared with two extreme clinical scenarios (i.e., all patients were treated or none of them were treated). This further validated the good performence and high value of this model in practical clinical work.

This study has some limitations. This is a single-center study, the sample size and the representativeness of the sample might be insufficient. Our study was conducted from 2016 to 2022, and we initially collected 1550 patients, and finally, after rigorous inclusion–exclusion screening, the final sample size was 1178, which was much larger than the minimum sample size requirement of constructing a risk prediction model (323 patients)^35^. Therefore, we considered that the results of our study can be applied well in clinical practices. Although this prediction model has been set up with a validation set for internal validation, an external validation with a larger sample size and multiple centers would be helpful to demonstrate the feasibility of this model in order to better generalize it. We intend to conduct more multicenter investigations to improve the sample's representativeness and applicability of the study results in future studies.

In conclusion, this study showed that age over 55 years, diabetes, ascites, spontaneous bacterial peritonitis, abnormal glutamate aminotransferase, and abnormal blood potassium concentration are independent risk factors(predictors) for the development of hepatic encephalopathy in patients with cirrhosis, and these six indicators are very meaningful for identifying the risk of developing hepatic encephalopathy in patients with decompensated cirrhosis. The risk prediction nomogram model based on the above risk factors can effectively and conveniently predict the risk of developing hepatic encephalopathy in patients with decompensated cirrhosis. This model can help clinical healthcare professionals to timely and early identify patients at high risk of developing hepatic encephalopathy, so as to intervene early and prevent the disease progression in time.

## Data Availability

The datasets used and/or analysed during the current study available from the corresponding author on reasonable request.
